# Round the Hospitals

**Published:** 1919-02-08

**Authors:** 


### ROUND THE HOSPITALS.

### TRUTH TESTS FOR M.P.s.

(By Request.)

We have been asked persistently of late to enable members of Parliament to test for themselves the relative authority and credit which attaches to the statements of the opponents of the College of Nursing. The enemies of the College have taken to themselves, as most nurses know, high-sounding names with the thinnest of tissue paper to rest on. One of their umbrellas is "*The Matrons' Council of*

**ROUND THE HOSPITALS**—(*continued*).

"*Great Britain and Ireland*," and another "The "National Council of Trained Nurses of Great "Britain and Ireland." The latter has what it describes as a "Grand Council," formed of "Dele- "gates of Affiliated Societies of Trained Nurses." In the last few weeks, the Treasurer of "The "National Council of Trained Nurses of Great "Britain and Ireland" announced that the balance to its credit at the bank was £22 4s. 11d. The Honorary Treasurer of "The Matrons' Council of "Great Britain and Ireland" produced a financial statement which showed a balance in hand of £10 16s. 10d. at the end of the year. It will thus be seen that the claims they put forward to members of Parliament and the public, as well as to nurses, that these persons represent to any real extent British Nursing Opinion and should carry weight and authority, cannot be accepted as fact or truth. Those who are sufficiently interested to test the untruthfulness of the opponents of the College of Nursing should compare the Verbatim Report of the meeting on Nurse Registration held at 1 Wimpole Street on January 23—it will be found on pages 383 to 393 inclusive of The Hospital of the 1st instant—with a report of the same proceedings appearing on pages 68 to 72 of the *British Journal of Nursing* of the same date. Every member of Parliament interested in nursing questions owes it as a duty to himself and his reputation, that before he touches nursing matters he shall compare these two reports and be seized of the true position and facts. The omissions and misrepresentations in the *British Journal of Nursing's* account of the proceedings on January 23 may be welcomed. For they give members of Parliament evidence which should shatter any attempt of the enemies of the College of Nursing to subsist on make-believe and false pretences.

---

Sir Cooper Perry's able and clear statement, published *in extenso* in our last issue, of the case in favour of the College of Nursing Registration Bill ought to be carefully read by all nurses, for it gives in a nutshell the cause of the long delay which has taken place in bringing any considerable body of nurses to demand legislation. In explaining that the nurses of the best London and provincial hospitals are able to make a living on the reputation of the nursing certificates they hold from their own schools, and thus have at present comparatively little inducement to enter for or pay fees for any further qualifying certificate, or for registration itself, he puts his finger on the root cause of the apathy long displayed about Registration. Until the sympathy and support of the leading nurse-training schools were enlisted everything languished. As soon as this was accomplished the Register became of immeasurable value, and there was a rapid growth of interest all over the country.

---

There is room for far more vigorous spade-work in nursing affairs, so ingrain is the tendency to regard professional matters from the point of view of single self-interest. "What good is Registration going to do me?" cries the ignorant nurse, and is quite unconscious of the pitiful figure she cuts in imagining she can stand or fall alone. There is no need for despair. All this is part of the awakening process. While all were asleep no one thought sleep was ignominious. Many nurses are now awake, and they must set themselves to rouse the rest. A nurse of some years' standing walked into the College offices the other day and asked what it all meant. She had never heard of the College of Nursing till that moment! One by one the nurses have to be brought in, convinced of the existence of their own profession, instructed from the beginning in the things which belong to their higher interests. This is how the soil is turned over. None can do it better than nurses among themselves.

---

It is curious to watch how time brings about a turn of the wheel. Years ago it was the leading training-schools which fought shy of the registration principle. They could not trust its promoters. Many of the leading matrons opposed it because they could not tell where it would lead. They disliked the idea of a Nursing Council through which power would be placed in the hands of a few persons with whom they were not in sympathy. In vain the advocates of registration pleaded that all would be well once the machinery was set up. The opposing matrons dreaded the machinery might be manipulated, and held sternly aloof. Now, the case is reversed. It is the former registrationists who are holding back. They will not trust the imposing and united forces of the nursing profession. They are retreating inch by inch, contesting every foot of the ground. Their last plea is distrust. They will not trust nurses with the future of the nursing profession!

---

The King has awarded the Albert Medal to Sister Gertrude Walters Carlin and Staff Nurse Harriet Elizabeth Fraser, both of the T.F.N.S., and to Sister Gladys White, of the B.R.C.S., in recognition of their gallantry in saving life at a casualty clearing-station in Belgium on October 1, 1918. The awards open up one of those hidden chapters of danger heroically encountered which have shed renown on the Nursing Services. It appears that a fire broke out in No. 36 Casualty Clearing-station at Ronsbrugge, in Belgium, at an early hour in the morning, when several of the patients were under the anæsthetic, and actually undergoing serious operations. The walls of the theatre were of wood. Suddenly, before any alarm of fire had been given, the electric light went out, dense clouds of smoke were perceived, and a second or two later the walls burst into flames. Without a moment's delay the three nurses, with the surgeons and assistants, carried the unconscious patients to a place of safety, and then returned to the other burning wards to help with the remaining patients. The air was filled with fumes and with flying fragments of glass and steel from the continual explosion of either bottles or nitrous oxide cylinders.

---

Too little is known of the East African campaign and of the dangers and hardships gallantly en-

**ROUND THE HOSPITALS—(*continued*).**

countered by the Army and the Medical and Nursing Services throughout its long and stubborn course. Lions and serpents took their part in it. Sundry kinds of disease and death awaited the Forces at every turn of the event. A pestilential climate had to be purged of its death-dealing qualities. That the nursing sisters participated in many of the hardships and terrors of the campaign in this savage country goes without saying. Their presence was an unspeakable comfort and support. Lieut.-General Sir J. L. van Deventer, K.C.B., C.M.G., Commanding in Chief, East African Force, has issued a list of those who have rendered excellent service, and recording his appreciation. It includes:—*Imperial Section.* — *Q.A.I.M.N.S.R.*: Staff Nurse E. Bridgens, Sister A. Donald, Sister G. E. Holmes, Staff Nurse M. M. Wood, Sister M. Macdevett, A.R.R.C. *T.F.N.S.*: Staff Nurse E. M. Pett, Sister E. Webbley. *South African Section.*—*S.A.M.N.S.*: Sister V. E. Bartlett, Probationer Nurse Bartmann, Probationer Nurse Miss G. Boden, Sister M. Cartlidge, Staff Nurse J. B. Davis, Probationer Nurse G. E. Holmes, Staff Nurse D. Leonard, Sister A. E. A. Lowe, Probationer Nurse W. Niven, Staff Nurse A. Slater, Staff Nurse H. L. Waldron, Probationer Nurse E. B. Williams. *E.A.M.N.S.*: Assistant Matron A. V. Stewart. *Norforce.—North Rhodesia Medical Corps*: Sister A. R. Stenhouse.

Lt.-General Sir G. F. Milne, K.C.B., K.C.M.G., D.S.O., Commander-in-Chief, British Salonika Force has recorded his appreciation of the gallant conduct and distinguished services rendered by the following members of the Nursing Services attached to the expedition:—*Q.A.I.M.N.S.*: Acting Matron A. B. Cameron, R.R.C. *Q.A.I.M.N.S.R.*: Staff Nurse L. Barnes, Staff Nurse M. H. Bebb, Staff Nurse H. Campbell, Sister H. M. Habgood, Staff Nurse E. Jamieson, Staff Nurse L. Levitt, Staff Nurse D. Marshall, Staff Nurse M. R. Noakes, Staff Nurse M. E. Riekie, Staff Nurse C. E. Seymour, Sister M. O. Simpson. *T.F.N.S.*: Matron V. A. Billing, R.R.C.; Sister E. A. Duston, A.R.R.C.; Staff Nurse J. A. Fraser; Sister E. Hall, A.R.R.C.; Staff Nurse E. K. Jarvis; Staff Nurse J. Moore; Sister E. Nichols; Staff Nurse (Acting Sister) H. Powell-Evans. *A.A.N.S.*: Sister I. Bowman, Staff Nurse I. A. Burns, Sister M. J. Readford.

Two nursing sisters have gained the Military Medal, under the King's approval, for distinguished service in the field. Sister Helen E. Hansen. C.A.M.C., was in the enemy air raid at Etaples on the night of May 19-20, 1918. She was on duty in the operating-room throughout the severe bombardment, which lasted two hours, and is officially reported as "ready for any duty," and exhibiting qualities of coolness and courage. Sister Beatrice MacNair, C.A.M.C., during the same air raid, carried on her duties throughout the night without interruption, showed great solicitude for the patients in her ward, and was wholly unmindful of her personal safety. It has seldom been our good fortune to record on one day distinctions earned by so many nursing sisters in different localities.

Lady Ampthill has received a very warm letter of congratulation from Lieut.-Colonel E. W. Fiske, M.C., U.S.A., on the work done by members of Voluntary Aid Detachments in nursing and caring for patients at the American Base Hospital, Dartford. Most efficient aid also, he states, has been rendered in the kitchen, the laundry, and the motor department. In thanking the members for the assistance given to the United States Army, he records that it will be "something to be told, wherever we are, as proof of the cordiality, generosity, and nobility of British womanhood." It is something, indeed, to have been instrumental in setting the name of our countrywomen a little higher than before.

The Committee of the Royal Devon and Exeter Hospital has recently considered the salaries paid to its nursing staff, and has unanimously agreed on an improved scale, to take effect from Christmas 1918. Under this scale the highest salary is paid to the night sisters, who are to receive £55 per annum, rising by £5 increment to £65. The day sisters receive £50 to £60; probationers £15, £20, £30. On the private staff nurses in their first year receive £40, in their second £50, and in their third year £60. We trust the salaries of the matron and her assistant have also undergone a substantial increase.

The first steps towards co-ordination in infant welfare schemes were taken on January 28, when an informal meeting was held at the premises of the British Red Cross Society in Pall Mall, at which representatives of various societies interested in child and infant welfare were present. A small informal committee was appointed to draft a preliminary scheme for subsequent discussion. There can be no doubt that a lead is required in this direction. Many individuals and municipalities are undertaking, rather lightheartedly, infant welfare work of the most delicate description. We fear in many cases nurses half trained, or not trained under the right conditions, are placed in charge, and the result is much wasted effort and discouragement.

That war bonuses would remain as additions to salary has been confidently expected. We see that the Christ Church Guardians have formally adopted the recommendation of the Finance Committee that this shall take place in respect of the nursing staff as from January 1, and we anticipate that this example will be generally followed. The Committee has also recommended that, in recognition of the extra work imposed by a shortage of staff, the following gratuities be paid, subject to the approval of the Local Government Board: Superintendent nurse, £10; first assistant nurse, £7 10s.;

**ROUND THE HOSPITALS—(*continued*).**

all nurses with over six months' service, £5; other nurses, £3. A cordial letter of thanks has been sent to the superintendent nurse, acknowledging with gratitude the extra work performed.

The Women's Guild of the General Hospital, Bristol, held its annual meeting and show of work on January 24, when 3,186 articles were on view, comprising everything needed in the institution. The subscriptions amounted to £258. Mrs. Granger Stock, who has held the post of honorary secretary to the Guild during the past eighteen years, is now retiring, and this was made the occasion for presenting her with a gold wristlet watch, set with pearls, from her fellow members.

The Manchester and Salford Sick Poor and Private Nursing Institution held its annual meeting in January, and it was then announced that the Nurse Cavell Memorial Fund amounted to £1,525. It is held by the Corporation upon trust to pay the income to the honorary treasurer for the purpose of providing an additional nurse or nurses for the sick poor of Manchester and Salford. A special appeal was made not long ago for £20,000 to endow the institution, and of this £13,404 has already been subscribed. Under an agreement with the War Pensions Committee discharged sailors and soldiers will now be attended by the nurses. The importance of having an endowment for carrying on the work of nursing the sick poor in a town like Manchester is easy to comprehend. The normal staff is seventy-six, but, owing to various causes, there are at present no less than thirty-two wanting, a very serious condition of affairs indeed.

The new scheme brought out by the National Housing and Town Planning Council contains a highly original suggestion, which, if carried into effect, would do much to lessen the labours of institutions. It is that local authorities should be empowered to build houses specially designed for families, members of which are suffering or are likely to suffer from tuberculosis. Many a highly hopeful case returns home after open-air treatment only to relapse on account of the unsuitable accommodation offered in the ordinary bedroom of town and country. We would go a step further, and suggest that all new houses should be designed in such a way as to enable the tenants to put in a good resistance to germs of every description. Even the model dwelling and the new type of cottage err badly in respect of both window space and of the number of panes which can be opened. The cottager who reserves his window-sill for the geranium could keep his flowers in bloom and yet admit fresh air if he were only provided with top lights.

The influence of mind upon body is aptly illustrated by the discovery that the new wave of influenza corresponds in time, like the last one, with a notable break in the weather. A writer in the *Times* insists that any circumstance which tends to detract from a man's personal sense of well-being favours the onset of disease. Hence the importance to nurses who are exposed in the course of their duties to disease germs of being additionally well cared for during an epidemic. An extra glass of hot milk or cocoa at a time when the vital forces begin to run low may keep the whole staff fit. The lesson for all institution managers is to re-inforce their workers and keep them up to the top of their form. Then extra work will be shouldered gallantly, for it is not only in East Africa, Salonika, and France that nurses have a brave heart. Incidentally, it would be interesting to know whether every institution has taken steps to equip the nurses in the pneumonia wards with face masks.

Ill-judged interference with domestic details in the nurses' home at Camberwell Infirmary has been responsible for bringing about a serious crisis among the nurses. Without consultation with the medical superintendent, Dr. Masterman, or presumably with the matron, the visiting Commitee inaugurated a drastic system of economy as regards heating, curtailed the hours when hot baths could be had and cut off other privileges. The medical superintendent pointed out that he, and not the Guardians, was responsible for the health of the nurses, and that he would not be answerable for the consequences. His protest was ignored. The nursing staff then took the matter into their own hands and addressed a strong protest to Mrs. Phillips, the chairman of the Committee, declaring that unless their grievances were redressed and the conditions of living rendered bearable, they could no longer continue their duties. The letter was widely signed, and Mrs. Phillips, with two of her colleagues, at once consented to receive a deputation of nurses at the infirmary. The complaints were speedily found to be justifiable. Orders were given for the heat to be put on in the nurses' bed-rooms and for hot baths to be available for night nurses returning from leave, with other slight privileges previously withdrawn. The incident shows a strange disregard of common sense and ordinary courtesy on the part of the visiting committee in attempting to re-organise domestic matters unsupported by those responsible for the nurses' welfare or by adequate knowledge of the hardships involved in their new scheme.

**☛ It is our invariable practice to pay for all items of news which we may publish. The minimum payment is 5s. Paragraphs of about twenty lines in length—that is to say, of 150 words or less—are preferred. Contributions should be addressed to The Editor, The Hospital, 28 and 29 Southampton Street, Strand, London, W.C. 2, and, to be in time for the current week's issue, should reach him by the first post on Mondays.**

